# Fortune favours the brave: composite first-person narrative of adolescents with congenital heart disease

**DOI:** 10.1136/bmjpo-2017-000186

**Published:** 2017-11-17

**Authors:** Giovanni Biglino, Sofie Layton, Lindsay-Kay Leaver, Jo Wray

**Affiliations:** 1 Bristol Heart Institute, Bristol Medical School, University of Bristol, Bristol, UK; 2 Cardiorespiratory Division, Great Ormond Street Hospital for Children, NHS Foundation Trust, London, UK

**Keywords:** cardiology, adolescent health

## Abstract

**Background:**

An interdisciplinary framework including a narrative element could allow addressing lack of awareness or excessive anxieties and teasing out divergences between patients’ health status and their expectations. This could be particularly relevant for adolescents with congenital heart disease (CHD).

**Objective:**

To develop a collective narrative ensuing from a creative activity involving adolescents with CHD, in order to explore their health perceptions and expectations.

**Design:**

Artist-led workshop process supported by a multidisciplinary team.

**Setting and participants:**

Young people with CHD (n=5, age 17–18 years, two men) were involved in the creative process, which encouraged them, over two sessions, to elaborate imagery relating to their uniqueness as individuals and their hearts. On top of creative activities (including self-portraits, embossing, body mapping and creative writing), participants were also shown their hearts in the form of cardiovascular MRIs and three-dimensional (3D) models manufactured by means of 3D printing.

**Methods:**

A composite first-person narrative approach was adopted to handle the emerged phenomenological descriptions and creative outputs, in order to shape a unified story.

**Results:**

The composite first-person narrative highlighted themes central to the patients, including their interpretation of medical references, their resilience and their awareness of anatomical complexity.

**Discussion and conclusions:**

Exploring the narrative of adolescents with CHD can offer unique insight into the way they view their hearts at a crucial stage of their care. An artist-led creative workshop supported by a multidisciplinary team can be a valuable approach to collect such narratives from patients and begin exploring them.

**What is already known on this topic?**

- Patients with cardiac disease transitioning from the paediatric to the adult centre need to take on increasing ownership for their condition.
- New technologies can provide unprecedented insight into the human body, but the narrative element remains a neglected dimension of data.

**What this study hopes to add?**

- We present a first-person composite narrative from the perspective of adolescents with congenital heart disease (CHD).
- An immersive artistic workshop can allow adolescents with CHD to express imagery relating to their individuality, incorporating elements of their medical history.
- An interdisciplinary framework including a narrative element could contribute towards teasing out divergences between patients’ health status and their expectations.

## Introduction

Unearthing narratives and ‘honouring the stories of illness’^1^ are essential for developing a holistic approach to medicine. As discussed in the literature on *co-creation*, the management (from the carer’s perspective) and self-management (from the patient’s perspective) of a condition rely on practical and moral choices that are profoundly unique,^2^ and it has been advocated that a narrative approach could be illuminating with regards to adopting technological innovations to improve patients’ care. An interdisciplinary framework including the narrative element could thus potentially allow improvements, such as addressing lack of awareness or excessive anxieties and teasing out divergences between the patients’ health status and their expectations. Furthermore, while new technologies can dramatically improve our insight into the human body, with the most sophisticated imaging techniques or new technologies such as three-dimensional (3D) printing, they cannot exhaust its meaning.^3^ In other words, how is this medical reality reconciled with the *experiential component*? The narrative element has indeed been suggested as an essential yet neglected dimension of data.^4^ And its inclusion, particularly based on an artistic participatory approach, could prove beneficial to identify both collective and unique responses in a population of interest.^5^

More specifically, in the context of adolescent patients growing up with congenital heart disease (CHD), we have previously discussed how an immersive artistic workshop was conducive to generating imagery allowing young people with CHD to express the uniqueness of their condition, and that this process can give them the opportunity to explore their individuality within a group sharing a similar medical condition and life experience, incorporating elements of their medical history.^6^ Here, we discuss how a collective narrative can be developed, ensuing from this kind of creative activity, and what its value might be for patients and health professionals.

## Materials and methods

### Participants

Five young people with CHD (age 17–18 years, two men) were involved in the workshop process. All participants were under follow-up in a specialist cardiac transition clinic at a tertiary paediatric hospital and were invited to take part in the workshops on the basis of a primary diagnosis of CHD and availability of cardiovascular MRI data in order to create a 3D rendering of their heart (see Workshop process section). Their primary diagnoses included tetralogy of Fallot, total anomalous pulmonary venous drainage and transposition of the great arteries. Participants provided written assent/consent to be photographed during the workshop, for the conversations to be recorded, and for their creative outputs to be shared with the group and more widely. All patients were in the final 2 years of secondary education and were in the process of applying for university. They were accompanied in the process by an artist with long-standing experience in participatory practices, an adolescent clinical nurse specialist, a biomedical engineer and a health psychologist.

### Workshop process

All participants took part in two sessions, which were run 2.5 months apart. The first session explored the uniqueness of CHD with both artistic media and 3D printed models, while the second was more overtly focused on the heart as an organ.

As part of the process, during the first session (described in detail in Layton *et al*^6^, see Supplementary File), participants were led through activities including a blind self-portrait drawing exercise, a blind self-portrait sculpting exercise, a creative writing activity and a body map exercise. These activities enabled them to develop language and imagery personal to them and their perception of themselves, without explicitly focusing on their heart and their condition.

**Figure 1:**
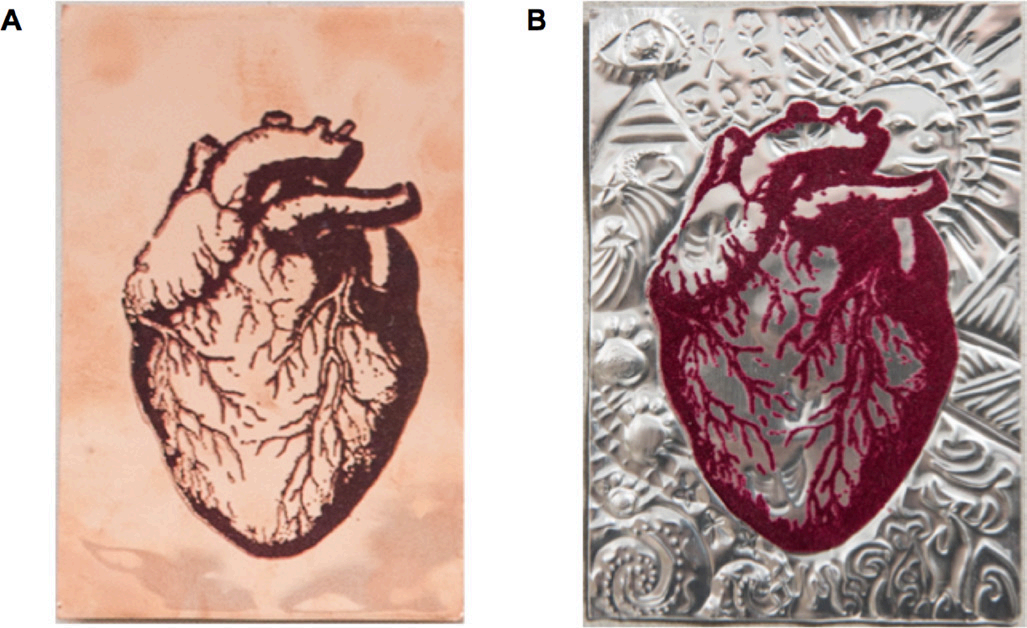
Participants completed an embossing activity using small A6 copper or aluminium plates with an anatomical drawing of the heart printed onto A. The embossed pieces incorporated elements of each participant’s personal imagery that was explored during the workshop. An example of embossed piece is shown in B.

During the second session, the group re-engaged with one another and the facilitators. During an opening exercise, participants revised the imagery that was developed during the first workshop. They then undertook an embossing activity, whereby they were given a small A6 size metal plate (either aluminium or copper, allowing for participants’ preferences, see figure 1A) with a velvet flocked anatomical heart screen-printed on it; participants were asked to incorporate the imagery that had emerged from the first workshop, in particular the body mapping exercise, to create unique embossed pieces.

A short creative writing exercise ensued, to further develop imagery relating to the self. Overall, across the two sessions, participants were asked to elaborate on how they saw themselves if they were: an animal, a colour, a weather/element, a building, a vegetable, a book/film, a childhood toy, a flower and a piece of clothing. This activity allows participants to brainstorm introspective images and they were encouraged by the artist facilitating the workshop not just to identify an image for each theme but to elaborate on the reason why they associated that specific image with themselves.

**Figure 2:**
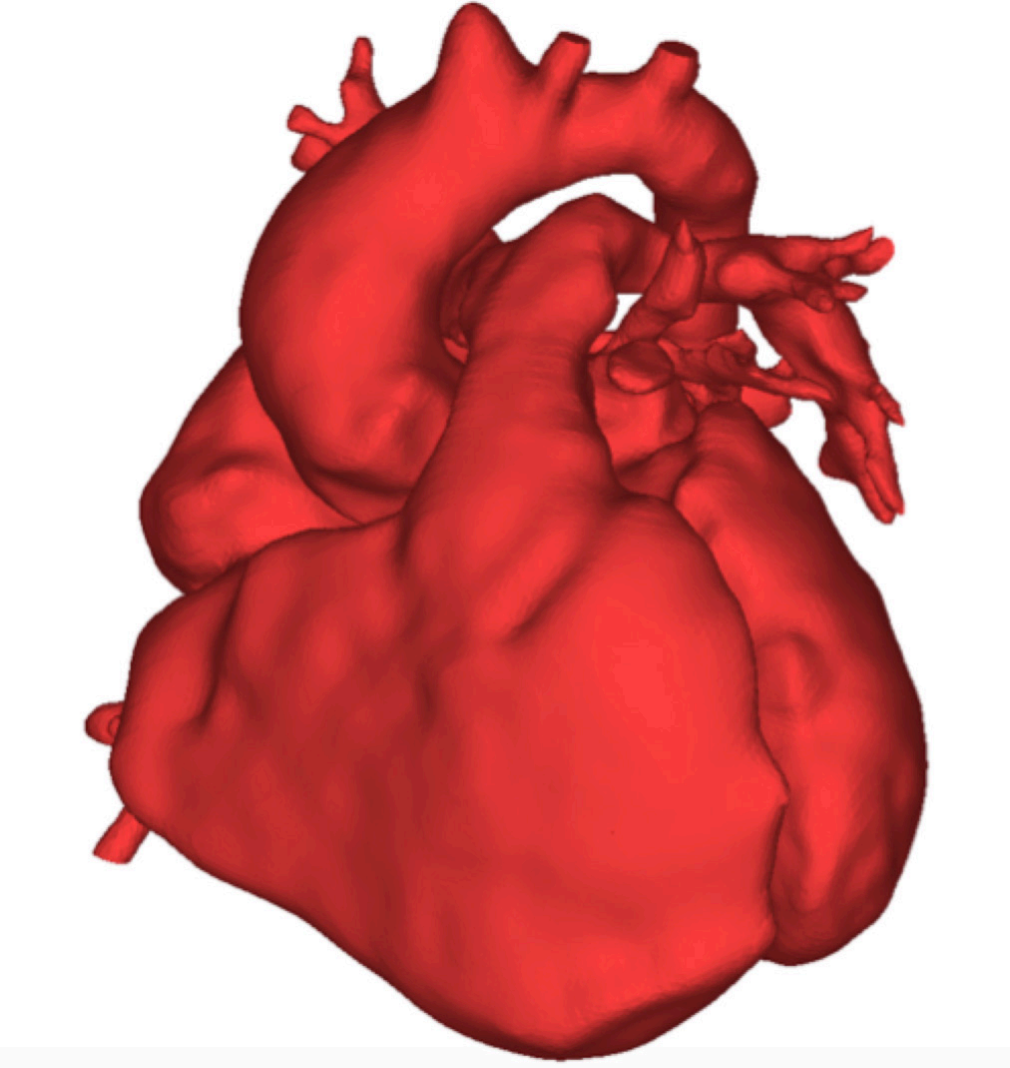
Participants worked on the outline of their own heart, which was reconstructed from three-dimensional (3D) data as part of their routine cardiovascular MRI scan. An example of 3D reconstruction is shown here. These patient-specific heart renderings were given to participants to trace in A4 size.

The final activity focused explicitly on the heart. Participants were given an A4 size printout of their own hearts. These were obtained by three-dimensionally reconstructing the cardiovascular MRI data for each patient, namely the whole heart sequence, according to validated methodology,^7^ and then printing the 3D rendering in two dimensions. An example is provided in figure 2. First participants were asked to outline their own heart using tracing paper and connect with its lines and forms. The outlines were then photocopied on thicker A4 sketching sheets and, as a final activity, participants created their heart design, having access to a range of paints and pastels, which enabled them to incorporate colour and text in the design if they so wished.

Throughout the process, participants were encouraged to speak about their images and drawings. The workshop was recorded for later analysis and reflection.

### Interpretation of findings: first-person narrative

A composite first-person narrative approach was chosen to handle the emerged phenomenological descriptions.^8^ The composite first-person narrative is a reflective story and results in a representation of the phenomenon amalgamating the voices of multiple participants. Initially, the narrative was independently developed by different authors (artist, engineer and psychologist), and in order to do so, they revisited audio recordings and notes from the workshops, as well as—importantly—visually assessing the artistic output of the workshop. The latter chiefly included the heart designs realised at the end of the second session, but took into account all the imagery that had emerged and had been discussed throughout the process. Prior to approaching the writing, the authors dwelled on the materials for approximately 18 months. A composite approach incorporating narratives unearthed through formative research allows the researchers to use factually realistic details and shape a unified story.^9^ Three authors (GB, SL and JW) developed a narrative independently and differences in the approach, tone and key elements to be included were then discussed prior to creating a merged version, which resulted in the final ‘composite’. This was then shared with the fourth author (L-KL) to further check the truthfulness of the representation. As such, there was not a dominant writer but the approach was considered as a group authorship. The final version of the composite narrative (presented in the Results section of this paper) was also shared via email with the workshop participants. They were invited to comment on whether elements of it reflected their own individual narrative and the feedback that we received from them indicated that this was indeed the case.

## Results

Participants engaged well in the workshop process overall. Two participants were taken aback by the final activity of working on their own heart printouts, but were guided by the artist through the activity and the resulting designs were rich in imagery. Importantly, all young people were willing to discuss elements of their artworks and elaborate on them. This sometimes happened not during a group reflection but rather during one of the activities, with the embossing exercise, for example, acting as a displacement activity allowing for participants to start elaborating on their imagery while slowly working on the metal plate. The group bonded well and the resulting element of peer support facilitated conversations around the creative outputs, which were indeed rich and detailed.

The outcomes of the workshop process allowed the research team to develop the composite first-person narrative of adolescents with complex CHD, which is presented below.

Is it red? That is what I thought it looked like when I was little.

My whole life has been defined by my heart—the good and the not so good. I knew that something was wrong with my heart, that it needed fixing, even when I was little. I remember people saying that there was a hole in it (a strange concept to grasp) and I imaged that they closed it by putting a plaster over it, and they also put some bandage around the aorta, as it too needed mending. Now I understand that it is much more complicated than that. It is something like a Rubick’s cube, a puzzle; something challenging, layered and complex, that maybe eventually cannot be done. People have tried to fix it but it cannot be fixed—it is unique and special but bits are in the wrong place. Sometimes I wonder: “Does it actually look like a heart?” because some of its parts are missing.

And over the years, my heart survived the trauma of being repaired. Today, it bears scars, which are a testimony to this trauma, but it survived, and I am so proud of it. Almost like a soldier, who has been wounded during the wars, and lived on, so now, together with the scars, it is decorated with medals, like a war hero. The medals represent its resilience, its strength and its achievements. It is my condition that defines who I am and fuels my drive and my determination to succeed, to grab life whenever I can. I have scars, my soldier has scars, each a wound that tells a tale of a different fight but each victory necessary for me to be here. The soldier is camouflaged to protect and conceal, returning each time from the battlefield, bloodied and sore, but the wounds of war heal, leaving a scar and a medal to cherish—a symbol of the bravery.

I know that the scars are always there, and they are black, like black lines, black marks. So the heart is red—and black. And then, sometimes I feel the colours start to change, gradually veering from cold to warmer and warmer shades. It’s still cold, but it’s warming up. And the warmer part is at its core. Colour has always been important, vital even. As a child I remember hearing about ‘blue blood’ and ‘red blood’, or people commenting on my blue lips and nails—not really blue, more like a purplish tinge. Little did I realise then that the colour I was indicated to others how well my heart was functioning. What colour am I today? If I was a colour it would be purple, that is how I see myself. The degree of purple tells me (and others) if I need to rest and how well I am feeling. But my colour changes, oscillating between blue and red, that process of freezing and warming like a frozen snowflake melting and being heated up by a burning fire. That is like my life—good days, bad days, days when I can do things and days when I can’t. And that is it—the puzzle and unpredictability of my life.

My heart is a survivor. It defines me, it shaped my life, challenging me and those looking after me but rewarding me and them too, at times, as obstacles are overcome and battles are won, living life to the fullest. It is different, it stands out from crowd, bearing its scars and its medals, and makes me stand out of the crowd too.

I also sometimes imagine that it is inscribed with words from comics that I read when I was little and books that I have read as I was growing up. So many things are important in my life—things that I keep close to my heart and that make me who I am. Comics, books, the theatre—all there as supports, comforters at times of stress, sources of strength, but also purely to be enjoyed. *A Midsummer Night’s Dream*, *Great Expectations*, the wooden sword I used to play with as a child, all mixed up.

My heart is unique, and strong, and fragile. And when I think of it I am reminded of a Latin saying that I learnt at school: ‘audentis fortuna iuvat’, fortune favours the brave. How true that is! There are times—I’m not going to lie—when I wish I didn’t have anything wrong with my heart, that I could be like a ladybug with the power to fly and be free of all problems, but my heart makes me who I am and leaves me with an overwhelming sense of pride and achievement. And yes, a feeling of good fortune.

## Discussion

CHD requires lifelong treatment and/or support, with a growing population of adults with CHD.^10 11^ Adolescence, when patients ultimately transition from the paediatric to the adult centre, is a particularly significant time in their care, which entails them ideally gaining independence, understanding of their condition, appreciation of complications and lifestyle adjustments and taking on increasing responsibility and ownership for their condition from their parents.^12–14^ Here, we suggest that an exploration of the narrative of young people with CHD could offer unique insight into the way they see their heart. Furthermore, as the way in which narratives are solicited from patients is important,^9^ we propose that a creative workshop led by an artist with participatory experience and supported by a multidisciplinary team can be a valuable way to begin exploring such narratives.

Having adopted a composite first-person narrative approach, it was possible to identify themes that are central to the patients that were involved in the creative process. These include:

1. The use of medical references: these comprise mainly scars, but also the idea of the ‘hole in the heart’, bandages, plasters and patches.
2. The resilience of these patients: images of strength and battle have clearly emerged, predominantly with one ‘soldier heart’ dressed in camouflage and decorated with medals, but also with the use of the Latin quote ‘Audentis fortuna iuvat’ (Fortune favours the brave).
3. The use of writing in the design: participants incorporated elements of writing, whether keywords or entire sentences.

Additionally, the use of colours was very unique and participants were given absolute freedom in choosing how to develop their design. Some used pastels and the results were denser and richer, some used watercolours resulting in more delicate and softer designs, while one participant focused on the design as an outline and eloquently included the piece of a jigsaw, suggestive of his personal view on surgical replacements and repairs that had taken place on his heart.

Variables including ethnicity, social status, level of education or type of professional occupation (including parental education) are known to affect neuropsychological outcomes in patients with CHD.^15^ In our study, the sample size was too small to evaluate differences in some of the key variables at play, but we note that participants were all at an equivalent stage in their education and engaged well during the workshop process. Furthermore, we would advocate that the artistic process contributed to creating a bond between participants which, qualitatively, was demonstrated by their high level of engagement, willingness to share their stories and returning for a second workshop. It is important also to consider character traits typical of a young generation that tends to be techno-savvy and collaborative^16 17^ in support of adopting a creative and visual approach in a workshop setting to explore patients’ narratives.

The workshop was framed as an artistic activity and not as art therapy. This is an important distinction, as the artist leading the workshop was focusing on using the creative tools to stimulate and hold the narratives. Participants’ benefit, nevertheless, could be inferred from the feedback received via email after the activities, mostly referring to the possibility of sharing their accounts and to the opportunity of doing it with peers who also have a form of CHD. One participant eloquently reflected on the approach being “very useful when going through the transition clinic” as “[i]t made me feel like I still mattered as opposed to feeling like I was being forgotten and passed on without much thought”, and that “[t]he work with the artist allowed me to actually reflect on what my condition meant to me and how it impacted me growing up; this was a good way to mark the transition into being an adult patient”.

The use of a composite approach does not diminish the individual voice and contribution. Each individual account contains unique elements and should in itself be respected and hailed as significant.^18^ A composite approach does not intend to dilute this uniqueness or suggest that singular images or expressions should be removed in an amalgamation of common traits. Rather, the composite approach was chosen as a way to protect individual stories and identities, by combining all of them into one. Indeed, it is suggested that this method could lead to a ‘more embodied’ understanding of the phenomenon being represented, conveying its wholeness.^8^

The exploration and assimilation of stories of illness has been advocated to lead to better understanding and, as a result, improvement of healthcare.^19^ Taking into account the patient’s narrative can provide not only biographic or social references that might not otherwise emerge, but also allow for insights into the patient’s development to surface.^20^ In our case, it was important to identify a theme of resilience and the contrasted feelings (eg, a scarred heart vs a heart that is warming up) in a population at a crucial stage of their care.

Taking into account the professionals’ perspective, it has been argued that the use of an artistic approach can lead to enriching the discourse of the practitioners.^5^ This has been beautifully elaborated by eminent narrative medicine scholar Rita Charon and described as a “Cézanne-like shift to the right or the left” that “gives […] sight of questions we are not wise enough to ask” to patients.^21^ In referring to the famous anecdote of Paul Cézanne painting over and over his subject of the Montagne Sainte-Victoire overlooking Aix-en-Provence having realised that just moving his sight a few inches to the left or to the right he was able to view his subject entirely afresh, Charon describes the subtle yet powerful insight that can be achieved by virtue of a narrative approach.

Such an approach can be complementary to the insight into CHD that can be nowadays gathered with advanced technologies such as exquisite medical imaging, refined computational modelling or 3D printing.^22–24^ It is complementary in that it allows us to go beyond crucial themes such as anatomy, function and complications, enabling us to start exploring the nature of health and the idea of pain and its sources, such concepts which have been referred to as central ontological and existential questions on individual uniqueness and human worth.^25^

## Conclusion

A composite first-person narrative from the perspective of adolescents with CHD was created following an artistic workshop during which patients explored imagery relating to their individuality and their heart.
